# Mesenchymal Stromal Cells and Tissue-Specific Progenitor Cells: Their Role in Tissue Homeostasis

**DOI:** 10.1155/2016/4285215

**Published:** 2015-12-28

**Authors:** Aleksandra Klimczak, Urszula Kozlowska

**Affiliations:** L. Hirszfeld Institute of Immunology and Experimental Therapy, Polish Academy of Sciences, Rudolfa Weigla 12 Street, 53-114 Wroclaw, Poland

## Abstract

Multipotent mesenchymal stromal/stem cells (MSCs) reside in many human organs and comprise heterogeneous population of cells with self-renewal ability. These cells can be isolated from different tissues, and their morphology, immunophenotype, and differentiation potential are dependent on their tissue of origin. Each organ contains specific population of stromal cells which maintain regeneration process of the tissue where they reside, but some of them have much more wide plasticity and differentiate into multiple cells lineage. MSCs isolated from adult human tissues are ideal candidates for tissue regeneration and tissue engineering. However, MSCs do not only contribute to structurally tissue repair but also MSC possess strong immunomodulatory and anti-inflammatory properties and may influence in tissue repair by modulation of local environment. This paper is presenting an overview of the current knowledge of biology of tissue-resident mesenchymal stromal and progenitor cells (originated from bone marrow, liver, skeletal muscle, skin, heart, and lung) associated with tissue regeneration and tissue homeostasis.

## 1. Introduction

Many human organs and tissues, including skin, liver, muscle, pancreas, lung, adipose tissue, placenta, bone marrow (BM), and peripheral blood, as well as others, contain an undifferentiated population of tissue-resident cells facilitating tissue repair and tissue remodeling during the life-time. These cells are characterized by specific properties: self-renewal capacity, the ability to give rise to descendant progenitor cells, multipotency, and the capability to differentiate into a variety of cell types specific for particular tissues. Tissue-resident stromal cells usually are localized in a specific local tissue microenvironments that maintain and control a particular type of cells or their progenitors for differentiation and maturation.

However, stromal cell function of many organs is diminished with age leading to reduced regenerative potential of all organs [1]. In the literature, different types of tissue-resident mesenchymal stromal cells (MSCs) are described; however, it is not clear if these cells are specific only for tissue regeneration from which they originate or whether their heterogeneity allow them to differentiate into various types of cells. MSCs isolated from various tissues share a number of nonhematopoietic cell markers including CD29, CD44, CD73, CD90, CD105, and MHC class I antigens. Nonimmunogenic properties of MSC are permitted by the lack of MHC class II antigens and lack of costimulatory molecules CD40, CD80, and CD86. These characteristics make MSCs promising candidates for new therapeutic strategies in transplantation and regenerative medicine.

Cells bearing MSC characteristics have been isolated from different organs and tissues of the human body including BM, adipose tissue, skin, muscle, tendon, bone, brain, liver, kidneys, lungs, spleen pancreas thymus, synovial membrane, and umbilical cord [2]. Intensive studies on MSCs are performed from years; however, the location and role of native MSCs within their own tissue environment* in vivo* are not fully explained, mainly because of the lack of specific markers allowing their precise recognition [3]. In self-renewing organs, stromal cells reside in specific niches that constitute the microenvironment in which tissue-specific progenitor cells are maintained in a quiescent state. After activation signal delivery, progenitor cells proliferate and migrate to the sites of injury where they differentiate and acquire the mature phenotype [4]. Tissue-specific progenitor cells niche homeostasis is regulated by the division of progenitor cells, which maintain the quantity of primitive and committed cells within the tissue [5].

MSC originated from different tissue locations exhibited many common characteristics; however, some markers are distinguishing for differentiation potential of these cells. This review is introducing the similarities and differences between MSCs originated from different type of tissues based on their surface markers and their regenerative potential in organs where they reside and their multipotential ability to differentiate into other lineages.

## 2. Mesenchymal Stem Cell of Bone Marrow Origin

Up to date, MSCs originated from adult bone marrow stroma are the best characterized mesoderm-derived stromal cells with multipotent differentiation capacity. The term of MSC was introduced by Caplan in 1991 as a type of adult stem cells with natural potential to differentiate into diverse mesenchymal cell types including osteoblasts, chondrocytes, adipocytes and others [6]. Historically, MSCs were isolated for the first time from the bone marrow by Friedenstein as a fibroblastic precursors with unknown anatomical location in the BM environment [7]. These cells were characterized by plastic adherent capacity with fibroblast-like morphology, extensive proliferation ability, and clonal expansion as confirmed by colony-forming unit fibroblast assay (CFU-F). Moreover, heterotopic transplantation of BM cells into different immunoprivileged site, including renal capsule, resulted in ectopic bone formation suggesting that osteogenic precursors are present within BM environment.

Since that time, extensive research on MSCs of bone marrow origin was performed to characterize biology and surface epitopes of MSCs. MSCs are heterogenic populations and express variety of surface epitopes including integrin receptors (CD29, CD49*α*), cell adhesion molecules (CD44, CD54, CD58, CD62L, CD105, CD106, CD146, and CD166), enzymes (CD39, CD73), growth factor receptors (CD140b, CD271, CD340, and CD349), intermediate filaments (nestin, vimentin, desmin, and neurofilament), and embryonic antigens (SSEA-1), but none of these molecules are specific for BM-derived MSCs (Table 1) [2, 8]. Isolation of MSCs based on STRO-1 [9], antinerve growth factor receptor CD271 [10, 11], or cell adhesion molecule CD146 expression [12, 13] documented their heterogeneity and clonogenic capacity of these cells. However, further studies documented that MSCs isolated based on CD271 and CD146 surface markers constitute two distinct populations of MSCs of BM origin and these subtypes may have different function during development and aging [14].

Heterogeneity of MSCs, different isolation procedure of native stromal cells, and diverse culture conditions were a reason for defining by Mesencymal and Tissue Stem Cell Committee of the International Society for Cellular Therapy minimal criteria which characterize human mesenchymal stem cells as (i) plastic adherent cells, (ii) with expression of CD73, CD90, and CD105 surface markers and lack of expression of hematopoietic markers CD34−, CD45−, CD14−, CD79*α*−, and HLA-DR−, and (iii) multilineage differentiation potential into osteoblasts, adipocytes, and chondroblasts [15]. If the above criteria are not completed, the term “mesenchymal stem cells” should be used for bone marrow-derived adherent cells or other MSC-like cells of different origin.

Extensive research describing MSC phenotype and biology has been performed on human BM-derived MSC* in vitro*, but there is still a little evidence on their phenotype in their natural* in vivo* environment. Recent studies on trabecular bone biopsy specimens documented the presence of cells with pattern of MSC antigen expression with different morphology and microanatomic localization [8]. Nonreticular stromal cells including round stromal cells and bone lining cells express CD73, CD140b, and CD271 antigens. Round stromal cells additionally express CD10, whereas bone lining cells are distinguished by neural ganglioside (GD2) expression. Reticular stromal cells such as fibroblastic reticular cells and adipose stromal cells (ASC) are overlapping CD10 and CD146 antigens and are distinguished by the presence of GD2 (on fibroblastic reticular cells) and CD73 (on ASC) [8]. In many studies, topography of MSCs in the BM environment is introduced as the cell lining the outer surfaces of blood vessels and perivascular cells and these cells express CD146 antigen [8, 16, 17]. MSCs sorted based on STRO-1+CD146+ phenotype expressed smooth muscle actin alpha (*α*SMA) which is also specific for pericytes [18]. Tormin studies introduced that CD146+/CD271+ BM cell fraction comprises both sinusoidal perivascular cells and cells residing in the BM environment, whereas bone lining MSC expressed CD271 alone [19]. All these observations suggested that MSCs residing in the medullary cavity, endosteum, and BM stroma represent distinct fractions of MSCs contributing to different progenitors development at the natural BM microenvironment.

In the BM environment, MSCs are involved in tissue homeostasis by contributing to hematopoietic stroma formation and regulatory molecules production including stem cell factor (SCF) and chemokine CXCL12, factors necessary for hematopoietic stem cell (HSC) niche regulation and maintenance. Downregulation of CXCL12 expression in reticular cells and osteoblasts results in HSC mobilization to the periphery and loss of B-cell progenitors, whereas the deletion of* Cxcl12* from stromal cells in perivascular region has influence on long-term HSC repopulating activity and common lymphoid progenitors [20]. However, perivascular HSC niche is more complex and is supported by other cell types including vessel endothelial cells, sympathetic nerves, nonmyelinating Schwann cells, macrophages, and osteoblasts, which in cooperation with perivascular MSC are responsible for self-renewal, proliferation, and trafficking of HSC, thus maintaining the pool of HSC [20]. Therefore, the level of CXCL12 expression on MSC originated from different HSC niches confirmed MSC diversity in the BM compartment and their influence on HSC and lymphoid progenitors activity.

Recent studies documented that stromal cell originated from different tissues (other than BM) showed significant differences in their differentiation and molecular phenotype and these findings suggest that stromal cells from other sources may be not able to substitute stromal cells of bone marrow origin [21].

## 3. The Liver Progenitor Cells

Regenerative potential of the liver is accomplished by resident hepatocytes and cholangiocytes when moderate liver injury occurs. However, self-renewal capacity of hepatocytes is limited when massive liver damage or partial hepatectomy takes place. Under these certain conditions, liver stromal/progenitor cells in humans [22, 23] and oval cells in rodents [24, 25], named for their morphological appearance as small cells with oval nuclei, can participate in liver regeneration. Human hepatic progenitor cells are bipotent precursors of hepatoblasts and cholangioblasts and reside at ductal plates in fetal liver and in canals of Hering in the vicinity of the portal triads of acini in adult livers [23]. They express specific marker EpCAM (*epithelial cell adhesion molecule*) allowing for their immunoselection (Table 1). EpCAM positive cells characterize high clonogenic activity for above 150 population doubling. Moreover, pluripotency of EpCAM positive cells and the ability to differentiate into biliary progenitors and hepatoblasts permitted self-renewal capacity of these cells. Except EpCAM, hepatic progenitor cells express CD29, CD133, and NCAM (CD56) molecules, and they are negative for hematopoietic markers (CD34, CD45, CD38, and CD14), for endothelial cell markers (VEGFR, vWF, and CD31), and for mesenchymal markers defined by authors as CD146, desmin, and *α*-smooth muscle actin. However,* ex vivo* clonogenic expansion of EpCAM positive cells revealed the presence of mesenchymal “companion” cells, which penetrate the colonies and were found throughout them. The mesenchymal “companion” cells represent two distinct populations: angioblasts positive for VEGFR, vWF, CD31, and CD117 (c-kit) and hepatic stellate cells that expressed CD146+, desmin, and *α*-smooth muscle actin. Additional rigorous immunoselection for EpCAM+ cells proved that paracrine signaling from mesenchymal “companion” cells is essential for EpCAM+ cells survival [23]. Presumably, among mesenchymal “companion” cells, pericytes (CD146+, CD90+, and CD140b+), normally localized around periportal blood vessels in human fetal and adult liver, contribute to clonogenic potential of EpCAM cells [26]. Studies on rodent model introduced that EpCAM is expressed on oval cells and on cholangiocytes, while TROP2 associated protein, a member of EpCAM family, is expressed exclusively in oval cells, indicating that TROP2 is a valuable marker for oval cells characteristics. TROP2 expression, upregulated in oval cells in injured liver, increases the possibility to modulate and/or augment the intracellular signaling of EpCAM to support proliferation and migration of oval cells into liver parenchyma [27].

Oval cells, recognized as facultative progenitor cells in adult liver that normally reside in the portal area of the liver, are proliferative quiescent. After severe injury of the liver, oval cells become activated and migrate into liver parenchyma and differentiate into hepatocytes and cholangiocytes. However, the origin of oval cells is controversial, and studies documented that oval cells are of bone marrow origin [28]. In severe liver injury, hepatocytes upregulate expression of SDF-1*α*, a potent chemoattractant for hematopoietic cells CXCR4+. Oval cells express CXCR4, the only receptor for SDF-1*α*. Interaction of SDF-1*α*/CXCR4 is essential to initiate activation of oval cells, when hepatocyte proliferation is impaired, and maintain stem cell niches through the control of progenitor cell migration by possible recruitment of a second wave of bone marrow origin progenitor cells to the injured side of the liver [25, 29].

**Figure 1:**
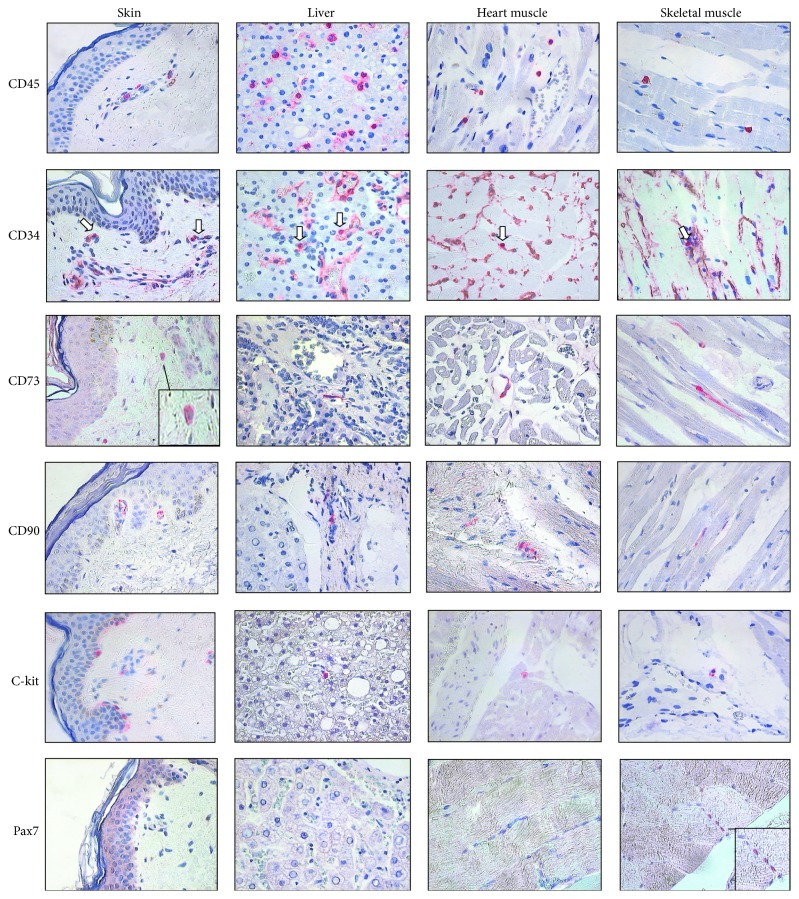
Tissue distribution and the phenotype of tissue-resident stem cells characterized by immunocytochemistry for CD45, CD34, CD73, CD90, c-kit, and Pax7. Tissue samples were collected from skin, liver, heart, and skeletal muscle. Immunostaining for CD45 and CD34 (arrows) illustrated the presence of cells of hematopoietic origin in the tissues. Note CD34 positivity on the vessel endothelial cells. Common feature of tissue localized stem cells was expression of CD73, CD90, and c-kit. Skeletal muscle progenitor cells exclusively express transcriptional factor Pax7. CD73, CD90, and c-kit were expressed on single stem cells of examined tissues and were localized in specific tissue compartments: in the basal layer of epidermis, the epithelium of adnexal structure of the skin, the periportal area of the liver, between the basal lamina and sarcolemma of myofibers of the muscle, and were connected to myocytes and fibroblasts in the cardiac niches.

The hepatic stellate cell represents the fraction of liver-resident cells with star-like morphology, located between liver sinusoidal endothelial cells and hepatocytes. Perisinusoidal stellate cells represent MSC of the liver and regulate essential hepatic physiological and pathological processes. During normal conditions, stellate cells are quiescent and have low proliferation rate, but, after liver injury, these cells progressively activate and change their dormant phenotype for active myofibroblastic-like phenotype. Myofibroblastic-like phenotype is characterized by the expression of *α*-smooth muscle actin (*α*-SMA) and desmin intermediate filaments. Moreover, activated stellate cells express neural markers including glial fibrillary acidic protein (GFAP), nestin, and N-CAM. These observations indicate a possibility of neural origin of liver stellate cells. These cells express also CD271, known as p75NTRF (nerve growth factor receptor family), which is a marker for mesenchymal stromal cells and is used for their positive isolation. However, the cellular phenotype of primary hepatic stellate cells depends on their fetal or adult liver origin and is highly dynamic, time dependent, and culture conditions dependent. At early stage, culture fetal CD271 positive cells did not express *α*-SMA and CD90, but after longer cultivation these cultured CD271 cells exhibit strong expression of these markers. In contrast, freshly isolated CD271 cells from adult liver expressed all the markers of stellate cells [30]. However, both types of CD271 cells expressed phenotype characteristic for MSCs including CD73 and CD105 and were negative for hematopoietic markers CD34 and CD45. Our own studies on tissue-resident stromal cells documented tissue distribution of cells with self-renewal capacity in the liver, expressing CD73, CD90, and c-kit, and these cells are localized in the periportal area of the liver as illustrated in Figure 1 [31].

Thus, regenerative capability of human liver is not associated with one type of liver progenitor cells with regenerative potential. Rather cooperation between different types of stem cells of the liver is necessary to maintain hepatic cells integrity and homeostasis.

## 4. Skeletal Muscle Mesenchymal Progenitor Cell

Skeletal muscle, similar to the most of postnatal tissues, contains naturally occurring pool of resident adult progenitor cells maintaining regenerative potential of skeletal muscle. The principal progenitor cells responsible for muscle regeneration are satellite cells, a quiescent bipotent tissue-specific cell population located between the basal lamina and sarcolemma [32]. Activation of satellite cells is triggered by muscle injury and is controlled by proximal signals from muscle niche, microvasculature, and inflammatory cells [33], as well as systemic factors [34]. Activated satellite cells act as stromal/progenitor cells contributing to the repair of damaged myofibers, or they are able to generate new myofibers following cell division and fusion with each other or with the existing myocytes. Moreover, satellite cells have the ability to replenish a reserve pool of tissue-resident progenitor cells in skeletal muscle via self-renewal capacity [35]. Quiescent satellite cells express CD34, CD56, and Myf5 surface antigens and paired box transcription factor Pax7; however, expression of CD34+ declined during differentiation into myoblasts [36]. Our own studies proved that MSC markers, CD73 and CD90, were expressed on single stem cells of examined skeletal muscle and were localized in the specific tissue compartments between the basal lamina and sarcolemma of myofibers of the muscle [31]. Moreover, skeletal muscle progenitor cells, but not progenitor cells present in the skin, liver, or heart exclusively express transcriptional factor Pax7 (Figure 1).

Satellite cell pool is relatively stable during the life; however, it may differ in specific muscle. It has been suggested that satellite cells consist of two distinct populations, one responsible for muscle regeneration, but their number is decreased with age, and the second which is activated in response to severe muscle injury and remains at constant amount throughout life [1, 37].

In addition to satellite cells, a variety of tissue-resident progenitors existing in skeletal muscle plays important role in the maintenance of tissue homeostasis [32]. Myogenic potential of nonsatellite progenitor cells was identified in a cell population residing in the muscle interstitium in the neonate [38]. These cells demonstrate multilineage potential and belong to mesenchymal progenitor/stromal cells (MSCs) as confirmed by broad range of gene expression common to MSC [39]. These muscle progenitor cells are characterize by the expression of CD34, stress mediator PW1, but they are negative for Pax7 (PW1+/Pax7− interstitial cells, PICs). Studies showed thatthese cells contribute to new myofibers formation and satellite cells generation as documented* in vitro* when cocultured with myoblasts or* in vivo* when transplanted into regenerating muscle environment. However, PW1+/Pax7− populations are negative for endothelial markers as proved by CD31 negative staining [38].

Another muscle-resident population of nonsatellite progenitor cells is bipotent fibro/adipogenic progenitors (FAPs) localized in the muscle interstitium and neighboring to muscle-associated blood vessels. These cells are phenotypically CD31−/CD45− and strongly express PDGF*α* and vimentin, markers associated with mesenchymal progenitors [40]. The majority of FABs (over 90%) have adipogenic capacity. However, these cells differ from PICs as they do not demonstrate direct myogenic potential. Mesenchymal FAPs progenitors, but not PW1+ cells, contribute to muscle regeneration by paracrine factors secretion of IL-6, IGF-1, and Wnt1 which markedly augmented myoblasts to terminal differentiation [41, 42].

Myogenic potential was also confirmed in endothelial-like mesodermal progenitors with pericytic features [43]. Pericytes, located within the basement membrane of vessels, in the human skeletal muscle represents myogenic precursors distinct from satellite cells. Muscle-resident pericytes are negative for myogenic markers including Myf5, MyoD, and MyoG. They are identified by alkaline phosphatase expression (AP) and they express neuroglial 2 proteoglycan (NG-2), platelet-derived growth factor receptor *β* (PDGFR*β*), and smooth muscle actin alpha (*α*SMA). Pericytes from the muscle stimulated* in vitro* are capable of myogenic differentiation.* In vivo* studies, on mouse muscular dystrophy, documented that pericyte transplanted into scid-mdx mice colonize host muscle and generate muscle fibres expressing human dystrophin [43]. Subsequent studies demonstrated that proportion of pericytes are capable to fuse with myofibers during early postnatal period and contribute to myogenesis.

Muscle-resident mesenchymal stromal/progenitor cells constitute heterogenous population of cells with diverse differentiating capability and play important role in tissue homeostasis. Most of them, like satellite cells, PICs, and pericytes, have direct myogenic differentiation capacity* in vivo*, whereas mesenchymal progenitors FAB/MSC effectively support myogenesis by paracrine growth factors secretion. Thus, effective regenerative potential of damaged skeletal muscle is associated with collaborative interactions between multiple heterogenous muscle progenitor cell types residing in the tissue.

## 5. The Skin-Derived Multipotent Stromal Cells

The presence of cells with regenerative potential in the skin can be attributed to maintain skin homeostasis and response to damage. Skin consists of epidermis and dermis layers, which are under steady regeneration process and contain a number of cells originating from mesoderm and ectoderm [44, 45]. Self-renewal capacity of the epidermis and hair follicles is dependent on precursor cells that exist in the epidermis, the dermal papillae, and the bulge. The presence of progenitor-like cells or MSCs in the skin was confirmed by the identification of several types of adult skin stromal or progenitor cells localized in both layers of the skin including dermal stromal cells and epidermal stromal cells [20, 45, 46]. Moreover, skin-derived precursors localized in several other skin structures such as hair follicles, blood vessels, sensory receptors, and nerve endings contribute to regeneration process and maintenance of the skin integrity. Isolated endogenous skin-derived precursors have the ability to proliferate for many passages with unspecialized phenotype, but under specific conditions they are able to differentiate into specific cell types including a neuroectodermal and mesodermal lineages. In the skin are also present different type of MSC, and their biological properties are different in cell culture. Adherent skin-origin MSCs are growing in the presence of serum, express markers specific for mesenchymal stem cell lineages CD73, CD90, and CD105, are negative for hematopoietic merkers including CD34, CD45, CD14, CD31, and HLA-DR, and are negative for nestin and positive for fibronectin, vimentin, and collagen type I. In contrast, skin-derived precursors in culture without serum form floating spheres and express nestin, the marker distinguishing them from plastic adherent cells [20, 45, 47]. Moreover, serum-free expanded floating spheres represent skin-derived precursors with limited mesodermal but higher neurogenic differentiation potential comparable to neural crest stem cells [45].

Diversity of human MSC of dermis origin was also confirmed in studies on mesenchymal progenitors isolated from foreskin samples [48].* In situ* analysis performed on skin samples revealed that MSC markers CD73, CD90, and CD105, as well as CD271 and SSEA-4, are expressed on different dermal cell types including endothelial cells (CD31+, CD34+) and leukocytes (CD45+). However, CD73, CD90, and CD105 positive cells lacking endothelial and leukocyte markers were also identified and these cells were characterized as a potential mesenchymal progenitor cells. Isolated dermal mesenchymal progenitors expressed surface markers similar to bone marrow-derived MSC. Dermal stromal cells represent very heterogeneous population, and except mesenchymal progenitors, within dermal plastic-adherent population, differentiated fibroblasts are present. Immunoselection of MSC based on CD271+ and SSEA-4 markers from adherent dermal cells confirmed their mesenchymal differentiation capacity and thus distinguished dermal MSC from differentiated fibroblasts. However, CD271+ cell population revealed higher adipogenic, osteogenic, and chondrogenic differentiation capacity compared to SSEA+ cells, which represent cell population of mesenchymal origin with differentiation potential limited to adipogenesis [48].

In the skin, taken from human thigh, we identified markers associated with phenotype of tissue-specific stromal cells, localized in the basal layer of epidermis and in the epithelium of adnexal structure of the skin (c-kit, CD90). CD73 positive cells were rather present in the perivascular area (Figure 1). These observations again proved diversity of tissue-resident stromal cells associated with their specific niche.

Thus, the skin, especially the foreskin and skin removed during aesthetic surgery, constitutes a selected biological waste material and can serve as an alternative source of progenitor-like cells for these MSCs of bone marrow origin, which may be applied for studies on tissue repair and cell-based therapy in regenerative medicine.

## 6. Cardiac Stem Cells

Human heart contains a population of primitive cells with self-renewal, clonogenic, and multipotent properties and these cells are able to differentiate into cardiomyocytes and coronary vessels. Resident cardiac progenitor cells represent heterogeneous population classified according to their biologic properties and surface markers for side population (SP), c-kit+ (CD117+), stem cell antigen-1 (Sca-1+), Islet 1+, SSEA-1+, and “cardiospheres” [49]. In the human myocardium, cardiac progenitor cells are localized within the cardiac niches composed of myocytes and fibroblasts, which represent the supporting cells, permitting maintenance of the balance between cardiac stem cell quiescence and activation [5]. Cardiac progenitor cells, with phenotype of CD73+, CD90+, and c-kit+, connected to myocytes and fibroblasts in the cardiac niches, were identified in our studies on tissue distribution of stromal/progenitor cells (Figure 1) [31].

The side population cardiac progenitor cells are heterogeneous and represent different subpopulations identified by expression of VE-cadherin, CD31, CD34, and Sca-1 and consist of vascular endothelial cells, smooth muscle cells, and mesenchymal progenitors including cardiomyogenic precursors. In rodents, SP cardiac progenitors were described as Sca-1+, c-kit+, CD34+, CD31−, and CD45− cells expressing cardiac specific transcriptional factor. After isolation and* in vitro* culture, SP cardiac progenitor cells acquired a cardiomyocyte phenotype documented by expression of sarcomeric proteins, troponin and *α*-cardiac actinin [49, 50]. Upon* in vitro* stimulation, these cells showed multipotent ability to differentiate not only into cardiomyocytes but also into typical neural crest-derived lineages including neurons, glia, and smooth muscle [51].* In vivo* studies on the rat model, documented the ability of SP cardiac progenitor cells to home damaged myocardium and to differentiate into cardiomyocytes and endothelial cells after intravenous infusion [52].

C-kit is a tyrosine kinase receptor for the stem cell factor primarily described on the hematopoietic stem cells of bone marrow origin [53]. A distinct resident cardiac stem cell population supporting cardiac regeneration, positive for c-kit, and negative for blood lineage markers CD34−, Lin−, and CD45− was reported for the first time by Beltrami et al. [54]. Subsequent studies confirmed the potential of c-kit positive cardiac progenitor cells in reducing infarct size and improving cardiac function after myocardial infarction [55]. Isolation and* in vitro* expansion of c-kit positive cells from cardiac tissue revealed differentiation potential to cardiomyocytes as confirmed by the expression of cardiomyocyte markers including *α*-cardiac actinin, cardiac myosin, desmin, and connexin [54, 55]. However, as reported by Tallini et al., c-kit positive cells act as cardiac progenitors until the neonatal phase, but in the adult myocardium they are rather responsible for neoangiogenesis [56]. C-kit+CD45− cells isolated from human cardiac biopsies coexpress endothelial progenitor cell markers CD31, CD34, CXCR4, and FLK-1, indicating further differentiation into endothelial cells [57]. Recent observations introduced the theory that c-kit positive cells constitute two populations, where the high c-kit+ cells work as cardiac progenitors and the low c-kit+ population might function as MSC [58]. Pluripotency of c-kit positive cells was confirmed by the differentiation ability into adipocytes and skeletal muscle myocytes.

Hypoxia favors cardiac progenitor cell quiescence, while normoxia is necessary for their activation and balance between hypoxic and normoxic cardiac progenitor cells may be present in young heart, whereas defects in tissue oxygenation occurring in the old myocardium may disrupt homeostatic control. Very recent studies reported that in senescent myocardium an increased number of quiescent c-kit positive cardiac progenitor cells with intact telomeres that cannot reenter the cell cycle are present, whereas myocyte repair is controlled by dividing cardiac progenitor cells with shortened telomeres. This observation suggests that a pool of functionally competent cardiac progenitor cells, nested in hypoxic niches in the senescent myocardium, can promote myocyte regeneration after activation by stem cell factor [59].

Sca-1 positive cells within myocardium represent heterogeneous subpopulation of cardiac progenitor cells based on the different subset of coexpressed stem markers. Cardiac progenitor cells expressing Sca-1+CD31+ and lacking the blood cell lineage markers c-kit, FLT-1, CD45, and CD34 negative were identified in adult murine myocardium [60]. These cells can differentiate into cardiomyocytes with the expression of structural cardiac genes. Sca-1 positive cells stimulated with oxytocin expressing c-kit, CD45, and CD34 generated beating cardiomyocytes, whereas Sca-1+CD45− cells in the same conditions revealed multipotent differentiation capacity into osteogenic and adipogenic lineages [61].

Islet-1 positive cells are considered as true cardiomyocyte progenitors appearing during embryogenesis and contribute to the right ventricle and outflow tract, although, it is unclear whether these cells exist in adult myocardium [62]. Within myocardium, cardiac progenitor cells expressing stage-specific embryonic antigen-1 (SSEA-1) are present. These cells represent a population of an immature pool of embryonic progenitors that differentiate into myocardial and endocardial cells at the neonatal stage of heart development. It has been suggested that SSEA-1+ cardiac stem cells can give rise to more committed cardiac progenitors expressing c-kit and Sca-1 [63].

Resident cardiac progenitor cells are abundantly present within the myocardium in niches preferentially located in the atria and apex and in the ventricle and effectively preserve the integrity of the tissue in the physiological conditions. However, the number of resident cardiac progenitor cells might be insufficient to repopulate injured tissue after extensive myocardial infarction. This may suggest that inherent ability of the myocardium to regenerate damaged myocytes after myocardial infarction is insufficient. This may be explained by the action of detrimental factors such as (i) deprived oxygen delivery in the infarct area leading not only to the cardiomyocytes necrosis but also to the death of resident progenitor cells within the infarct site, (ii) and resident cardiac progenitor cells, which accumulate acutely in the border of the infarct and cannot migrate from the viable tissue to the injured site because their translocation to the damaged myocardium is hampered. This is associated not only with anatomical barrier (scar formation) but also with limited production of growth factors (hepatocyte growth factor, insulin growth factor, and stroma-derived growth factor) facilitating recruitment of cardiac progenitor cells to the site of injury, and with inflammatory milieu of the injured myocardium which may have a negative effect on cardiac progenitor cells viability and differentiation [64, 65].

Thus, autologous resident cardiac progenitor cells, isolated from the adult myocardium, may offer distinct advantages over other adult stem cells for the therapy of cardiovascular diseases as they are tissue-specific and precommitted to the cardiovascular lineages.

## 7. The Lung Stromal and Progenitor Cells

The lung is a conditionally renewing organ and turnover of airway epithelial cells is less than 1% per day in the steady state conditions, and this regenerative capacity of the lung is in contrast to the continuously renewing tissue, such as bone marrow, with the ability to generate approximately 10^9^ hematopoietic cells daily. However, following severe injury, self-renewing potential of stromal and epithelial progenitor cells of the lung increases rapidly and compensatory growth of multipotent cells warrants proper regeneration of the lung [66]. Within the lung many diverse epithelial cell types exist and they are distributed in several different regional microenvironments along the pulmonary tract. Many studies on mouse models and a smaller number of literature reports on human lungs describe presumed populations of adult endogenous airway and alveolar epithelial progenitor cells; however, characterization and classification of these cells into a hierarchy are still controversial [67].

The organization of endogenous stromal and epithelial progenitor cells in the adult lung is specific for their regional distribution and function along the proximal-distal axis of the airway tree. The proximal part of the airway comprises the cartilaginous trachea, lined by columnar pseudostratified epithelial cells with submucosal glands, and includes basal, secretory, ciliated, and neuroendocrine cells. Basal cells represent progenitor/stromal cells of bronchiolar epithelium and are characterized by the expression of nerve growth factor receptor (NGFR), p63, cytokeratin-5, cytokeratin-14, and aquaporin 3. After isolation and* ex vivo* culture, they formed clonal structures positive for ciliated and club cells (known as Clara cells) [68, 69]. A population of basal cells can migrate from the bronchiolar niche into damaged alveolar epithelium and proliferate to repair alveolar region [69].

The distal part of the airway is lined with columnar epithelial cells and comprises different population of cells including club cells, ciliated cells, goblet cells, and neuroendocrine cells [66]. During epithelial homeostasis, club cells can self-renew and generate ciliated cells, whereas ciliated cells do not have the ability for self-regeneration [70, 71]. Within the club cells, residing along the distal axis of the airway tree, a distinct population of cells known as variant club cells is present and they are located at the bronchoalveolar duct junction. The variant club cells with self-renewal potential and differentiation capacity into club cells are able to repair bronchiolar epithelial cells after naphthalene injury [71]. Another population of distal airway stromal and progenitor cells is rare population of cells called bronchioalveolar stem/progenitor cells [66]. Bronchioalveolar progenitor cells are positive for the stem cell marker Sca-1, positive for EpCAM, and negative for hematopoietic (CD34, CD45) and endothelial cell markers (CD31) [72].* In vitro* studies documented that bronchioalveolar progenitor cells are able to differentiate into bronchiolar and alveolar colonies and have self-renewal capability. Moreover, their number increases after bronchiolar injury, and this suggests their role in tissue regeneration [73].

Terminal part of the lung constitutes alveoli with specific alveolar progenitor cells, which differentiate into surfactant-producing alveolar type II cells and gas-exchanging alveolar type I cells [71]. A population of alveolar progenitor cells, expressing laminin receptor *α*6*β*4 integrin, is located in the alveolar epithelium and is capable to contribute to airway and alveolar tissues regeneration in experimental model after parenchymal injury [74].

Resident lung mesenchymal stromal cells constitute a key element of epithelial progenitor niches along the proximal-distal axis of the airway tree [71, 72, 75]. The lung mesenchymal stromal cells secrete FGF 10, a critical factor necessary for directing differentiation in the developing lung [71]. Moreover, it has been documented that lung mesenchymal stromal cells, EpCAM negative and Sca-1 positive, cocultured with lung epithelial progenitor cells (EpCAM positive), support their proliferation and differentiation and generate colonies including airway, alveolar, or mixed lung epithelial cell lineages [75].

Regional stromal and progenitor cells such as submucosal gland/duct progenitor cells, basal cells, variant club cells, bronchioalveolar stem/progenitor cells, and alveolar progenitor cells that reside in distinct niches of the respiratory tract are responsible for the maintenance of specific epithelial cell lineages integrity in the specific region of the airways. Different populations of tissue-resident stromal and progenitor cells are involved in region-specific homeostasis and tissue repair after the injury of the lung. Thus, homeostasis of the lung is a highly coordinated process of proliferation and differentiation of lung stromal and progenitor cells and requires a balance between immune regulation and promotion of tissue regeneration.

## 8. Summary

Multipotent MSCs reside in specific tissue niches composed of cells creating specific microenvironment for tissue-resident progenitor cells and facilitate them to maintain tissue homeostasis. Niche cells provide signals which regulate and control the balance of self-renewal and differentiation capacity of stem/progenitor cells residing in them. The niche also controls stem/progenitor cell division and activity to preserve cancer formation. The balance of progenitor cell quiescence and activity is a hallmark of a functional niche and is regulated by internal (e.g., DNA damage) and external signals leading to self-renewal and differentiation of progenitor cells.

MSC can be easily isolated from various tissue sources, expanded in the culture, and appropriately differentiated under proper conditions. Depending on their tissue of origin, MSCs are predisposed to give rise to the type of tissue cells from where they are coming. Thus, MSCs from adult human tissues are ideal candidates for tissue regeneration and tissue engineering. However, MSCs do not only contribute to structurally tissue repair, but MSCs possess potent immunomodulatory and anti-inflammatory effects, and through direct cell-cell interaction or secretion of various bioactive factors they may have an effect on local tissue repair by modulation of local environment.

## Figures and Tables

**Table 1:** Heterogeneity of tissue-resident stem/progenitor cells.

Adult stem cell source	Stem/progenitor cells	Tissue distribution	Isolation marker	Phenotype of mesenchymal cells of tissue origin	Selected references
Bone marrow	(Mesenchymal stem cells)	Bone marrow stroma	No specific marker	CD10, CD29, CD44, CD73, CD90, CD105, CD140b, CD146, CD271+, GD2, SSEA-1, and STRO-1	[2, 8, 15]
	Pericytes	Sinusoids of the bone marrow	STRO-1+/CD146+ CD271+/CD146+	CD10, CD29, CD44, CD73, CD90, CD105, CD140b, CD146, CD271+, STRO-1, and *α*SMA	[17–19]

Liver	Hepatic stellate cell	Perisinusoidal space between sinusoids and hepatocytes	CD271	CD73, CD90, CD105, CD271, *α*-SMA, desmin, GFAP, nestin, and N-CAM	[30]
	Hepatic stem cells	Ductal plates in fetal liver and in canals of Hering in adult livers	EpCAM	CKs 8, 18, and 19, CD29 CD133, CD44, EpCAM, and NCAM (CD56)	[23, 27]
	Pericytes in human liver	Periportal blood vessels	CD146	CD146, NG-2, CD90, CD73, CD105, CD140b, and vimentin	[26]

Muscle	Satellite cells	Between the basal lamina and sarcolemma of myofibers	Pax7	CD34, NCAM (CD56), Pax7, and Myf5	[32, 36]
	Progenitor cells PICs	Muscle interstitium	PW1+/Pax7−	CD34, PW1+	[39, 40, 42]
	Fibroadipogenic progenitors FAP/MSC	Muscle interstitium adjacent to myofiber associated blood vessels (distinct from muscle pericytes)	PDGFR*α*	CD34, Sca-1, PDGFR*α*, and vimentin	[39–41]
	Pericytes	Basement membrane of muscle vessels	PDGFR*β*	AP, NG-2, CD146, PDGFR*β*, and *α*SMA	[39, 42, 43]

Skin	Adherent cells (cultured with the presence of serum)	Basal epidermis, dermal papillae, and bulge	CD271/SSEA-4	CD73, CD90, CD105, and CD271 fibronectin, vimentin and collagen type I	[45, 47, 48]
	Floating spheres (cultured without serum)		Nestin	CD73, and CD90, CD105, CD271, nestin, fibronectin, and vimentin	[45, 47]

Heart	Cardiac progenitors	Within the niche composed of cardiomyocytes and fibroblasts	C-kit (CD117)	C-kit (CD117), CD73, CD90, CD34, Sca-1, SSEA-1, VE-cadherin, and Islet-1	[49, 50, 52, 60]

GD2: neural ganglioside; NG-2: neuroglial 2 proteoglycan, SSEA-1: stage specific embryonic antigen-1; *α*SMA: smooth muscle actin alpha; CKs: cytokeratins; AP: alkaline phosphatase; Sca-1: stem cell antigen-1.
